# Atherogenic Lipid Ratios Related to Myeloperoxidase and C-Reactive Protein Levels in Psychotic Disorders

**DOI:** 10.3389/fpsyt.2020.00672

**Published:** 2020-07-10

**Authors:** Elina J. Reponen, Ingrid Dieset, Martin Tesli, Ragni H. Mørch, Monica Aas, Trude S. J. Vedal, Elisabeth Haug, Ole Kristian Drange, Nils Eiel Steen, Sigrun Hope, Attila Szabo, Sherif M. Gohar, Kirsten Wedervang-Resell, Srdjan Djurovic, Ingrid Melle, Pål Aukrust, Ole A. Andreassen, Thor Ueland

**Affiliations:** ^1^ NORMENT Norwegian Centre for Mental Disorders Research, Oslo University Hospital and University of Oslo, Oslo, Norway; ^2^ Division of Mental Health and Addiction, Acute Psychiatric Department, Oslo University Hospital, Oslo, Norway; ^3^ Department of Mental Disorders, Norwegian Institute of Public Health, Oslo, Norway; ^4^ Department of Acute Psychiatry and Psychosis Treatment, Innlandet Hospital Trust, Reinsvoll, Norway; ^5^ Department of Mental Health, Norwegian University of Science and Technology, Trondheim, Norway; ^6^ Department of Østmarka, Division of Mental Health Care, St. Olavs Hospital, Trondheim University Hospital, Trondheim, Norway; ^7^ Department of Neuro Habilitation, Division of Clinical Neuroscience, Oslo University Hospital, Oslo, Norway; ^8^ Department of Psychiatry, Faculty of Medicine, Cairo University, Cairo, Egypt; ^9^ Division of Mental Health and Addiction, Department of Psychiatric Research and Development, Oslo University Hospital, Oslo, Norway; ^10^ Department of Medical Genetics, Oslo University Hospital, Oslo, Norway; ^11^ NORMENT Norwegian Centre for Mental Disorders Research, Department of Clinical Science, University of Bergen, Bergen, Norway; ^12^ Institute of Clinical Medicine, University of Oslo, Oslo, Norway; ^13^ Research Institute of Internal Medicine, Oslo University Hospital Rikshospitalet, Oslo, Norway; ^14^ Section of Clinical Immunology and Infectious Diseases, Oslo University Hospital Rikshospitalet, Oslo, Norway; ^15^ K.G. Jebsen Thrombosis Research and Expertise Center, University of Tromsø, Tromsø, Norway

**Keywords:** CVD risk, dyslipidemia, inflammatory biomarkers, schizophrenia, bipolar disorder

## Abstract

**Background:**

Cardiovascular disease (CVD) is a major cause of premature death in patients with psychotic disorders, where dyslipidemia occurs frequently. In the pathogenesis of these serious mental disorders, a low-grade inflammation seems to be a possible contributor. Concurrently, systemic inflammation and its interplay with dyslipidemia is a central driver in the pathogenesis of CVD. We hypothesize that evaluation of atherogenic lipid ratios together with inflammatory markers reflecting different inflammatory pathways with relevance for atherogenesis, could give novel information on immune-related mechanisms involved in early CVD risk in patients with psychotic disorders.

**Methods:**

As a measure for CVD risk we calculated atherogenic lipid ratios using established sex-specific cut-offs: Total cholesterol/high-density lipoprotein; HDL-c (TC/HDL) and triglyceride/HDL-c (TG/HDL) were evaluated in 571 schizophrenia (SCZ) and 247 bipolar disorder (BD) patients, and in 99 healthy controls (HC). In addition, as a measure of low-grade inflammation, we measured fasting plasma levels of nine stable atherogenic inflammatory markers in patients (SCZ, BD) and in HC. The elevated inflammatory markers and CVD risk in patients, as reflected by TC/HDL and TG/HDL, were further assessed in multivariable analyses adjusting for comorbid cardio-metabolic risk factors.

**Results:**

A markedly higher proportion (26%–31%) of patients had increased TC/HDL and TG/HDL ratios compared with HC. Plasma levels of high-sensitivity C-reactive protein (hs-CRP) and myeloperoxidase (MPO) were higher (p<0.05, p<0.001) in patients with psychotic disorders than in HC, and hs-CRP and MPO were independently associated with atherogenic lipid ratios in the multivariable analyses.

**Conclusions:**

Our findings suggest that low-grade inflammation and abnormal neutrophil activation may cause increased CVD risk in patients with psychotic disorders. These mechanisms should be further examined to determine the potential for development of novel risk evaluation strategies.

## Introduction

Psychotic disorders are characterized by significant comorbid cardiometabolic risk (1, 2), and cardiovascular disease (CVD) mortality is elevated in patients with psychotic disorders. Compared with the general population, the risk of cardiovascular mortality is almost 2-fold in bipolar disorder and 2 to 3-fold in schizophrenia (3–5). Undiagnosed CVD prior to cardiovascular death is more common in psychotic disorders than in the general population (6).

Furthermore, dyslipidemia has frequently been reported in patients with severe mental disorders (1, 2). It is well known that second generation antipsychotic drugs (SGA) are associated with dyslipidemia and other metabolic side effects (7), but long-term antipsychotic treatment is contradictorily associated with reduced CVD mortality (8).

Atherogenic lipid ratios such as the total cholesterol/high-density lipoprotein; HDL-c (TC/HDL) and triglyceride/HDL-c (TG/HDL) have been shown to hold greater predictive value for CVD risk in individuals without symptomatic CVD than the isolated lipid parameters used independently (9, 10). Although increased TC and TG and reduced HDL-c have been reported (1, 2), these ratios have scarcely been investigated in psychiatric disorders.

It is well known that lipid accumulation together with low-grade inflammation lead to a chronic vascular remodeling and development of atherosclerosis in the arteries (11). An increasing number of novel inflammatory biomarkers that predict cardiovascular risk have recently been identified. These biomarkers are therefore relevant to investigate in patients with psychotic disorders, where a low-grade inflammation is a possible pathogenic contributor (12, 13).

Based on the emerging role of inflammation and its interaction with dyslipidemia in the progression of atherosclerotic disease, we hypothesize herein that evaluation of atherogenic lipid ratios together with inflammatory markers reflecting different inflammatory pathways with relevance for atherogenesis, could give novel information on immune-related mechanisms involved in the premature CVD risk in patients with psychotic disorders.

Our specific aims of this study were three-fold. Firstly, we evaluate whether the distribution of pro-atherogenic lipid ratios differ between a large cohort of patients with psychosis compared to healthy controls (HC). Secondly, we investigate whether the following inflammatory markers are dysregulated in patients with psychotic disorders versus HC:

- General down-stream markers of inflammation: High-sensitivity C-reactive protein (hs-CRP) (14) and glycoprotein 130 (gp130, a member of the interleukine-6 receptor family) (15).
- Markers of vascular inflammation, calcification, and endothelial function: Pentraxin 3 (PTX3) (16), osteoprotegerin (OPG) (17), and von Willebrand factor (vWF) (18).
- Markers related to fibrosis and extracellular matrix (ECM) remodeling: Galectin 3 (19) and Cathepsin S (20).
- Marker of neutrophil activation: Myeloperoxidase (MPO) (21).
- Marker of vascular apoptosis; Insulin-like growth factor-binding protein 4 (IGFBP4) (22).

Thirdly and lastly, we further investigate whether CVD risk as estimated by lipid ratios is associated with any upregulated inflammatory markers identified in the patient population. This study presents a detailed and thorough analysis of these three topics on a uniquely large cohort of patients with psychotic disorders.

## Methods

### Design and Participants

The current study is part of the ongoing Thematically Organized Psychosis (TOP) Study at the Norwegian Centre for Mental Disorders Research (NORMENT). The TOP Study includes patients from both outpatient clinics and hospitals in the Oslo, Trondheim, and Lillehammer regions in Norway. Inclusion criteria in the TOP Study are: diagnosis of severe mental disorder, age between18–65 years and ability to give written informed consent. The healthy controls (HC), between 18–60 years old, were randomly selected from statistical records (www.ssb.no) in the Oslo region. All participants have given informed written consent. The study was approved by the Norwegian Scientific Ethical Committees and the Norwegian Data Protection Agency.

### Sample

The sample used for the current cross-sectional study consists of 818 patients with severe mental disorders and 99 healthy controls with fasting blood samples available, all included in the TOP Study from 2002 until 2013. In this sample, 571 patients had a schizophrenia spectrum disorder (SCZ: schizophrenia, schizoaffective disorder, schizophreniform disorder and psychotic disorder not otherwise specified), while 247 patients had a bipolar spectrum disorder (BD: bipolar I, bipolar II and bipolar disorder not otherwise specified). The SCZ and BD groups combined are hereafter referred to as the “all patients” group.

Participants with non-fasting blood samples, autoimmune or inflammatory diseases, on-going cancer, on-going infections, C-reactive protein (CRP) >20 mg/L of any reason, insulin levels >400 pmol/L, or participants receiving treatment with immune modulating drugs were excluded.

### Clinical Assessments

All patients underwent a thorough diagnostic evaluation based on the Structured Clinical Interview in Diagnostic and Statistical Manual of Mental Disorders, 4^th^ Edition (DSM-IV) axis I Disorders (SCID-1). The Positive and Negative Syndrome Scale (PANSS), the Young Mania Rating scale (YMRS), and the Calgary Depression Scale for Schizophrenia (CDSS) were used for evaluations of the symtoms, and the functioning was assessed using the functioning score of the split version of the Global Assessment of the Functioning Scale (GAF-F). The inter-investigator diagnostic agreement has previously been evaluated to a satisfying level of 82%, with overall ĸ=0.77 (CI 0.60-0.94) (23).

Body mass index (BMI) (kg/m²) was calculated based on height and weight measured by standard methods. For weight digitally calibrated weights were used. Blood pressure was measured using a manual device under standard conditions. The HC were interviewed using the Primary Care Evaluation of Mental Disorders (PRIME MD) to assess current or previous history of severe mental disorder themselves or in their family.

### Blood Samples

The methodology used for drawing, processing and storage of blood samples have been described previously (24). Some of these biological markers have been reported previously by us in relation to other outcome measures (25–30).

#### Lipid Risk Factors

Plasma levels of total cholesterol (TC), triglyceride (TG), high-density lipoprotein (HDL-c), and low-density lipoprotein (LDL-c) were measured at the Department of Medical Biochemistry, Oslo University Hospital. TC, TG, and HDL-c were directly measured using an Integra 800 instrument from Roche Diagnostics, according to standard methods. LDL-c was calculated by Friedewald formula at the Department of Medical Biochemistry, Oslo University Hospital. This method changed during the study to direct measurement of LDL-c using an Integra 800 instrument from Roche Diagnostics.

#### Inflammatory Markers

Plasma levels of hs-CRP, gp130, PTX3, OPG, vWF, Galectin 3, Cathepsin S, MPO, and IGFBP4 were measured by enzyme immunoassays (EIA) in duplicate using commercially available antibodies (R&D Systems, Minneapolis, MN, USA) in a 384 format using a combination of a SELMA (Jena, Germany) pipetting robot and a BioTek (Winooski, VT, USA) dispenser/washer. Absorption was read at 450 nm with wavelength correction set to 540 nm using an ELISA plate reader (Bio-Rad, Hercules, CA, USA). Intra- and inter-assay coefficients of variation were <10% for all EIAs. For immunoassays blood was drawn using EDTA vials and the plasma was isolated the next working day and stored at -80°C. As plasma samples were collected over a long period, we evaluated potential degradation by comparing CRP measured continuously during samples collection and CRP measured during the bulk analysis of the other inflammatory markers. These CRP measurements correlated strongly (r=0.86) and both correlated similarly to time since sampling (r=0.17). Thus, the positive and similar association with sample time for CRP measured during sample collection and during bulk analysis argue against an effect of sample degradation during storage.

#### Insulin Resistance

Plasma levels of insulin and glucose were analyzed at the Department of Medical Biochemistry, Oslo University Hospital. Insulin was analyzed at the Hormone Laboratory by radioimmunoassay (RIA) using standard methods. Glucose levels were analyzed using standardized platforms from Roche Diagnostics. We estimated insulin resistance using the Homeostasis Model Assessment for Insulin Resistance (HOMA-IR) (31). As the calculation is valid only with insulin levels <400 pmol/L, participants with higher levels were excluded (N=11 patients).

Blood sampling was performed between 8 am and 11 am for most of the participants. All participants included in this study were fasting during blood collection.

### Medication

Information on the use of prescribed medications including antipsychotics, immune modulating medication and statins used by patients was assessed by clinical interview and hospital records. “Defined daily dose” (DDD) was calculated according to the World Health Organization (WHO) principles. We calculated the individual total DDD based on antipsychotic polypharmacy. The DDD is the assumed average maintenance dose per day for a drug used for its main indication in adults and provide a fixed unit of measurement independent of dosage form (http://www.whocc.no/atc_ddd_index/).

### Statistical Analyses

All statistical analyses were done using the SPSS software package for Windows, version 25.0 (SPSS Chicago. USA). All analyses were two-tailed with a level of significance set at p˂0.05. All skewed data was log-transformed prior to further analyses.

The cardio-metabolic risk was estimated using established atherogenic lipid ratios including TG/HDL and TC/HDL, with sex-dependent cut-offs established elsewhere (9, 10). Atherogenic lipid ratios were calculated, and enhanced cardio-metabolic risk was defined by (based on SI units, mmol/L): TC/HDL, male >5, female >4.5 (10); TG/HDL, male >1.53, female >1.09 (9). Differences in the proportion of individuals at risk between diagnostic groups were assessed using the chi-square test (unadjusted analysis). We then adjusted for demographics (age, sex, BMI) in logistic regression analysis (adjusted analysis).

Differences in levels of inflammatory biomarkers between HC and diagnostic groups (i.e., SCZ and BD) were analyzed by multivariate analysis of covariance (MANCOVA) adjusting for demographics (age, sex, BMI).

Finally, in inflammatory markers that were elevated in patients (hs-CRP and MPO), the unadjusted and multivariable adjusted estimated atherogenic risk (i.e., based on lipid ratios) within the total patient population (i.e., SCZ and BD combined), and within diagnostic groups (i.e., SCZ and BD analyzed separately), were assessed using logistic regression. In these analyses, TC/HDL or TG/HDL were included as the dependent variable and hs-CRP or MPO were included as covariates in block 1 (unadjusted analysis). Further adjustments for conventional CVD risk factors; age, sex, BMI, insulin resistance, smoking, systolic blood pressure, and anti-psychotic treatment (DDD), were performed in block 2 (adjusted analysis). An example of a fully adjusted model is shown in **Figure 2C**.

## Results

### Sample Characteristics

The clinical characteristics of the study population are shown in **Table 1**. The mean age and sex distribution in the patient group compared with HC were mainly the same when looking at the patient group as a whole. A subgroup analysis revealed that patients with BD were significantly older and more frequently female compared both to HC and patients with SCZ. The patients in general had significantly higher BMI, particularly so within SCZ. There was a higher proportion of non-European origin in patients compared to HC. The majority (78%) of patients received psychotropic drug treatment, with a higher DDD in SCZ than in BD.

**Table 1:** Demographics of the study population.

Clinical parameters	HC(n=99)	All patients(n=818)	BD(n=247)	SCZ(n=571)	Post hoc
	%(n)	%(n)	%(n)	%(n)	
Sex (male)	61.6 (61)	52.8 (432)	39.3 (97)	58.7 (335)	SCZ,HC>BD
Ethnicity (European)	98.0 (97)	82.2 (672)***	89.1 (220)	79.2 (452)	HC>BD,SCZ
Smoking status (daily use)	N/A	46.2 (367)	43.8 (106)	47.3 (261)	n.s.
Statin use	0 (0)	1.7 (14)	2.0 (5)	1.6 (9)	n.s.
	**Mean (SD)**	**Mean (SD)**	**Mean (SD)**	**Mean (SD)**	
Anti-psychotic treatment (DDD)	N/A	0.96 (0.96)	0.5 (0.77)	1.13 (0.97)	SCZ>BD
Age	30 (8)	31 (11)	34 (12)	30 (10)	BD>SCZ,HC
**Cardiometabolic risk factors**					
HOMA-IR	2.7 (1.5)	3.7 (2.6)**	3.4 (2.2)	3.8 (2.7)	SCZ>BD,HC
HDL-c(mmol/L)	1.51 (0.46)	1.36 (0.43)**	1.46 (0.48)	1.31 (0.40)	HC,BD>SCZ
LDL-c (mmol/L)	2.85 (0.90)	3.14 (0.95)**	3.02 (0.90)	3.19 (0.96)	SCZ>BD,HC
Total-c (mmol/L)	4.70 (0.93)	5.10 (1.07)**	5.06 (1.06)	5.11 (1.08)	SCZ,BD>HC
Triglycerides(mmol/L)	1.04 (0.44)	1.46(1.10)***	1.39 (1.13)	1.49 (1.08)	SCZ,BD>HC
BMI	23.9 (3.2)	26.3 (5.0)***	25.7 (4.4)	26.5 (5.3)	SCZ,BD>HC
**Symptom scores**					
PANSS total	N/A	58 (17)	46 (11)	63 (17)	SCZ>BD
CDSS total	N/A	5.5 (4.8)	4.8(4.9)	5.7 (4.8)	SCZ>BD
YMRS total	N/A	4.8 (5.2)	3.9 (5.3)	5.3 (5.0)	SCZ>BD
GAF-S	N/A	46 (13)	56 (12)	42 (11)	BD>SCZ
GAF-F	N/A	46 (12)	53 (13)	44 (11)	BD>SCZ

Analyzed with ANOVA for continuous variables and chi-square test for categorical variables. HC, healthy controls; BD, bipolar spectrum; SCZ, schizophrenia spectrum; n, number; DDD, defined daily dose; SD, standard deviation; HOMA-IR, homeostasis model assessment for insulin resistance; HDL-c, high density lipoprotein cholesterol; LDL-c, low density lipoprotein cholesterol; Total-c, total cholesterol; mmol/L, millimoles per liter; BMI, body mass index; PANSS, Positive and Negative Syndrome Scale; CDSS, Calgary Depression Scale for Schizophrenia; YMRS, Young Mania Rating Scale; GAF-S, Global Assessment of Functioning-symptoms; GAF-F, Global Assessment of Functioning- functions; N/A, not applicable; n.s., not significant; *p<0.05 **p<0.01 ***p<0.001 vs. healthy controls.

Evaluation of lipids revealed a dysregulated profile with higher TC, LDL-c, and TG and lower HDL-c in the patients, and these differences were most prominent in patients with SCZ. Furthermore, patients, and in particular those with SCZ, had a higher degree of insulin resistance as estimated by HOMA-IR compared to HC (**Table 1**).

### Pro-Atherogenic Lipid Ratios in Patients With Psychotic Disorders

To further evaluate the unfavorable lipid profile in patients with psychotic disorders, we calculated pro-atherogenic risk ratios based on lipid levels and assessed risk of CVD based on establish sex-specific cut-offs. Since indirectly measured LDL is derived from TC, and since TC/HDL ratio also is an established predictor of CVD risk, we focused the analyses on TC/HDL and TG/HDL. The patient group as a whole had a markedly higher proportion with enhanced TC/HDL (26.2%) and TG/HDL (30.5%) compared to healthy controls (9.2% and 8.2% for TC/HDL and TG/HDL, respectively). The unadjusted risk for an elevated TC/HDL was 3.35 [CI 1.65–6.80], p<0.001 in patients compared to controls, which persisted in age, sex, and BMI adjusted analysis, 2.69 [CI 1.29–5.60], p=0.008. Corresponding figures for TG/HDL were 4.77 [CI 2.27-10.01], p<0.001 in unadjusted and 3.16 [CI 1.47–6.80], p=0.003 in adjusted analysis.

**Figure 1:**
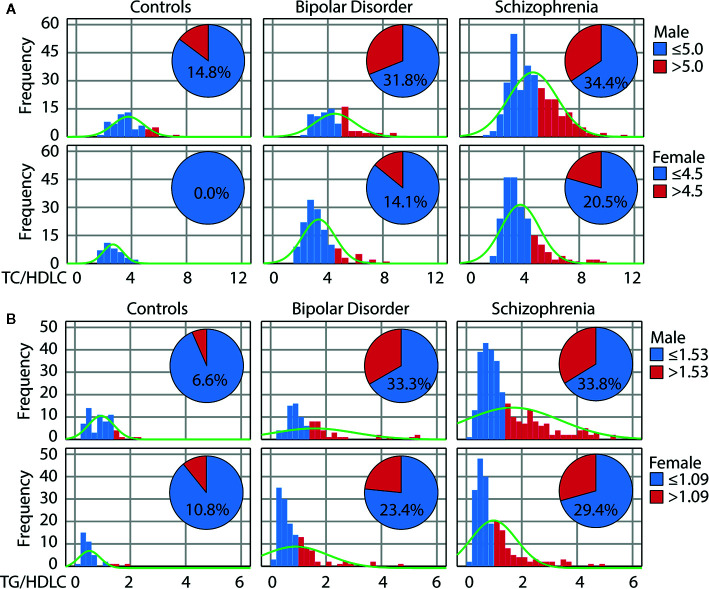
Increased cardiovascular risk in psychotic disorders as reflected by atherogenic lipid ratios. Distribution of the total cholesterol/high-density lipoprotein (TC/HDL) **(A)** and triglyceride/HDL-c (TG/HDL) **(B)** ratios in healthy controls, patients with bipolar disorder or schizophrenia according to sex. The pie diagram shows the number of individuals with ratios above the risk cut-off.

**Figure 1** shows the distribution of these lipid ratios according to diagnosis (BD, SCZ) and sex. Increased lipid ratios compared to HC were observed in both patient groups and sexes with a higher frequency in men and in patients with SCZ. Of note, the proportion of female patients with an elevated TG/HDL ratio was higher (23%–29%) than for TC/HDL (14%–21%), while these frequencies were similar in male patients (32%–34%).

### Inflammatory Markers in Patients With Psychotic Disorders

Patients as a whole were characterized by higher levels of hs-CRP (f=3.19, p=0.042) than HC in age, sex and BMI adjusted multivariate analysis of covariance (MANCOVA), shown in **Table 2**, with the highest levels in patients with SCZ (f=4.62, p=0.032) compared to HC. Circulating MPO as marker of neutrophil activation was markedly higher in patients; both in SCZ (f=16.01, p=7.1x10^-5^) and in BD (f=11.01, p=0.001) compared to HC. In contrast, we observed lower gp130 with a similar reduction in patients with BD (f=10.04, p=0.002) and SCZ (f=7.79, p=0.005) compared to HC. A similar association was observed for Galectin 3 in SCZ (f=40.5 p=4.2x10^-10^) and in BD (f=62.8, p=4.4x10^-14^) compared to HC, and for Cathepsin S in SCZ (f=6.91, p=0.009), and with the lowest levels in patients with BD (f=19.91, p=1.1x10^-5^), compared to HC. No significant differences were observed for PTX3, OPG, IGFBP4, and vWF, although low vWF levels were noted in patients with BD (f=5.94, p=0.015) compared to HC. Within the patient population (where smoking status was available), there were no significant difference in levels of inflammatory markers between smokers and non-smokers, except with respect to vWF where smokers had higher levels (95 AU in non-smokers vs. 108 AU in smokers, p=0.025).

**Table 2:** Level of inflammatory markers (MANCOVA) after adjustment for age, BMI, and sex.

	HC(n=99)	All patients(n=818)	BD(n=247)	SCZ(n=571)
hsCRP (mg/L)	1.78 (1.44, 2.19)	2.18 (2.02, 2.36)*	1.96 (1.70, 2.26)	2.29 (2.09, 2.51)*
PTX3 (ng/ml)	2.97 (2.53, 3.49)	3.01 (2.84, 3.19)	3.03 (2.72, 3.37)	3.01 (2.80, 3.23)
OPG (ng/ml)	1.32 (1.24, 1.40)	1.35 (1.32, 1.38)	1.36 (1.31, 1.42)	1.35 (1.31, 1.38)
vWF (AU)	82.6 (70.8, 96.6)	72.8 (68.8, 77)	67.3 (60.7, 74.7)*	75.3 (70.4, 80.7)
gp130 (ng/ml)	234 (224, 243)	217 (214, 220)***	214 (208, 220)**	218 (214, 222)**
GAL3 (ng/ml)	5.47 (4.56, 6.56)	2.67 (2.50, 2.86)***	2.33 (2.06, 2.63)***	2.84 (2.62, 3.08)*** †
CatS (ng/ml)	5.99 (5.57, 6.41)	5.35 (5.19, 5.50)**	4.99 (4.71, 5.26)***	5.51 (5.32, 5.69)** †
MPO (ng/ml)	179 (148, 218)	275 (256, 295)***	294 (259, 335)***	266 (245, 290)***
IGFBP4 (ng/ml)	161 (151, 170)	165 (161, 168)	166 (160, 173)	164 (160, 168)

Data are presented as estimated marginal means with 95% confidence interval. *p<0.05 **p<0.01 ***p<0.001 vs. healthy controls; †p<0.01 vs. bipolar disorder. MANCOVA, multivariate analysis of covariance; BMI, body mass index; n, number; hs-CRP, high sensitivity c-reactive protein; PTX3, pentraxin 3; OPG, osteoprotegerin; vWF, von Willebrand factor; gp130, glycoprotein 130; Gal3, galectin 3; CatS, cathepsin S; MPO, myeloperoxidase; IGFBP4, insulin-like growth factor-binding protein 4; mg/L, milligrams per liter; ng/mL, nanograms per milliliter; AU, arbitrary units.

### Association Between MPO, hs-CRP, and Atherogenic Lipid Ratios

**Figure 2:**
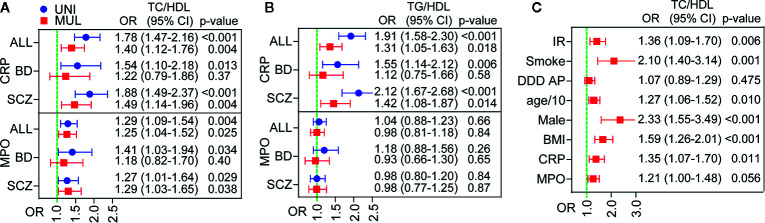
Association between inflammatory markers and cardiovascular disease (CVD) risk as reflected by atherogenic lipid ratios in patients with psychotic disorders. Univariate (blue) and multivariable (red) logistic regression of hs-CRP (CRP) (top) and myeloperoxidase (MPO) (bottom) as predictors of dysregulated lipid ratios TC/HDL**(A)** and TG/HDL **(B)**. Multivariable adjustment for total cholesterol/high-density lipoprotein (TC/HDL) is shown in **(C)** and included insulin resistance (IR), smoking, anti-psychotic treatment (DDD), age, sex, BMI, hs-CRP (CRP), and MPO. Odds ratios (OR) are expressed as log-transformed per SD change in marker.

Odds ratios (OR) were for these analyses based on log-transformed standardized values, and represent a one standard deviation (SD) increase in the analyzed marker. **Figure 2** shows unadjusted and adjusted logistic regression analysis for TC/HDL (**Figure 2A**) and TG/HDL (**Figure 2B**) ratios above the cut-off for CVD risk. Data are show for all patients and within diagnostic sub-groups (i.e., SCZ or BD).

As shown in **Figure 2**, unadjusted logistic regression analysis revealed a strong association between higher hs-CRP levels and CVD risk as reflected by elevated atherogenic lipid ratios, with OR 1.78 (95% CI 1.47–2.16, p<0.001) for TC/HDL (**Figure 2A**) and OR 1.91 (95% CI 1.58–2.30, p<0.001) for TG/HDL (**Figure 2B**). This association, although attenuated, remained significant in adjusted logistic regression analysis (OR 1.31 for TC/HDL and OR 1.40 for TG/HDL). Multivariable adjustment included insulin resistance (IR), smoking, anti-psychotic treatment (DDD), age, sex, BMI, hs-CRP (CRP), and MPO. The association was stronger in SCZ patients, where it remained significant in adjusted analysis with OR 1.49 (95% CI 1.14–1.96, p=0.004), while the association was not significant in BD following adjustment.

The association between MPO and CVD risk in patients was more modest and only present for the TC/HDL ratio with OR 1.40 (95% CI 1.12–1.76 p=0.004), but persisted after multivariable adjustment with OR 1.25 (95% CI 1.04–1.52, p=0.025). The association appeared at the same level in SCZ and BD, but was more attenuated by adjustment in BD and thus only statistically significant in SCZ in adjusted analysis OR 1.29 (95% CI 1.03–1.65, p=0.038).

As both hs-CRP and MPO were associated with increased TC/HDL, we also evaluated these markers together. **Figure 2C** shows that both proteins were associated with an increased TC/HDL ratio in multivariable adjusted analysis.

## Discussion

Our study, evaluating a large number of relatively young (mean age = 31 years) patients with psychotic disorders, shows that this patient group is characterized by a markedly higher CVD risk. This is reflected by a larger proportion of individuals with elevated atherogenic lipid ratios among patients with psychotic disorders compared to HC, in particular in male patients and in patients with SCZ. Plasma levels of hs-CRP, a reliable downstream marker of inflammation, and MPO, a marker of neutrophil activation and oxidative stress, were higher in patients with psychotic disorders, and independently associated with unfavorable lipid ratios in multivariable analysis adjusting for conventional CVD risk factors. These data suggest that low-grade inflammation in combination with an atherogenic lipid profile may cause increased CVD risk in patients with psychotic disorders.

Dyslipidemia has frequently been reported in patients with severe mental disorders, in both medicated and drug naïve patients (32). We demonstrated relatively stable lipid levels during longitudinal testing (5 years follow-up) in patients with schizophrenia and schizoaffective disorders suggesting that lipid abnormalities may be related to core disease mechanisms (33) in addition to antipsychotic medication (7). Regardless of cause, dyslipidemia and metabolic abnormalities are closely associated with an enhanced risk of cardiovascular outcomes. In this regard, atherogenic lipid ratios such as the TC/HDL and TG/HDL hold greater predictive value in individuals without symptomatic CVD than the independent lipid parameters (10). We were unable to find age and sex distributed reference data on the prevalence of increased ratios in the general population. However, the average TC/HDL ratio observed in healthy men and women in our study corresponded to the 50 percentile for 30 year-olds in a large contemporary population-based Dutch cohort study (34), suggesting that although limited in size the proportion of controls with increased lipid ratios in our study is comparable to similar European populations. This supports our data indicating a markedly enhanced CVD risk in this young population of patients with SCZ and BD, which may be related to the decreased life expectancy and plausibly increasing mortality due to cardiovascular causes in such patients (5, 35, 36). The higher proportion of patients with an elevated TG/HDL, compared to TC/HDL, (30.5% vs. 26.2%) in our study is compatible with the metabolic phenotype in these patients such as insulin resistance. Indeed, an increased TG/HDL ratio has been shown to closely correlate with insulin resistance in SCZ (37) as well as with the presence of small dense LDL particles which are particularly atherogenic (37, 38).

The interplay between dyslipidemia and chronic inflammation are hallmarks of atherosclerotic progression (39). CRP is the prototypical acute phase serum protein that rises rapidly in response to inflammation, but also represents the most robust marker of subclinical chronic inflammation. Chronic elevated levels have repeatedly been associated with persistent auto-inflammatory processes and an increased risk of future cardiovascular events in the general population (14, 40), and with CVD risk in patients with psychotic disorders (41–44). Our finding that elevated hs-CRP was associated with CVD risk as reflected by the TC/HDL and TG/HDL also in adjusted analysis including important cardio-metabolic risk factors, support these previous studies. Furthermore, this association was stronger and only significant after full multivariable adjustment in SCZ suggesting a stronger impact of inflammatory dysregulation on CVD risk in these patients. However, although hs-CRP by reflecting the general inflammatory burden related to important demographics such as insulin resistance and BMI (45, 46) represents a reliable risk marker of CVD, it is less useful in pinpointing specific dysregulation of inflammation that may be linked to CVD risk.

A major finding in our study was the markedly increased MPO levels in both SCZ and BD that were significantly associated with the TC/HDL ratio, also in adjusted analysis for SCZ. MPO is a reliable marker of neutrophil activation. These cells have recently received renewed interest with regard to atherosclerosis due to the variety of active substances they may release from granules, including reactive oxygen species promoting oxidation of lipoproteins, microparticles, and MPO that further are related to neutrophil extracellular traps (NETs). Aberrant neutrophil effector functions and increased circulating levels of inflammatory neutrophil effector peptides, such as alpha-defensins have been linked to SCZ pathogenesis (47) and may represent a bridging factor between atherosclerosis and inflammation (48). Indeed, elevated levels of MPO have been associated with coronary artery disease (CAD) (21) and predict risk in acute coronary syndromes (49). However, less is known on the role of MPO in individuals without symptomatic CVD, but epidemiologic studies demonstrate that MPO may predict future risk of coronary artery disease (50). Furthermore, elevated neutrophil counts have been reported in BD and SCZ (51–53) and based on the increased MPO levels observed in our study, it is tempting to speculate that abnormalities in neutrophil activation could represent an early event in the progression of CVD in patients with psychotic disorders. The stronger association with the TC/HDL compared to the TG/HDL, could suggest that the enhanced MPO is more directly linked to atherogenesis and not only a marker of this process.

We were unable to detect differences in a number of markers linked to vascular inflammation and CVD such as vWF (18), OPG (17), PTX3 (16), while levels of fibrosis and ECM remodeling markers such as Galectin 3 (19) and Cathepsin S (20) were lower, which may further support our hypothesis regarding dysregulated neutrophil effector functions and a consequent low-grade inflammatory “background”. These markers have repeatedly been associated with risk of adverse cardiovascular outcomes, but have mainly been investigated in patients with stable atherosclerotic disease. However, the association with CVD risk in patients without established CVD may be different than in patients with symptomatic CVD concerning both the strength and potentially the direction of association (54). Based on the relatively young age of our population, vascular inflammation and ECM remodeling, which accelerate during atherogenesis, may not yet be activated. However, we are not able to conclude with respect to this without following the same patient group over time.

## Limitations

This study had a cross-sectional design, making the causality described suggestive.

As fasting status can affect levels of the inflammatory markers, glucose, and lipid metabolism, we focused this study on fasting blood samples. This excluded 435 non-fasting controls and limited our control population to n=99, and gave uneven group size compared to the patient population (n=818). The relatively high portion of non-fasting participants in the initial control group was mostly due to their busy everyday lives, making it challenging to schedule blood sampling in the morning. These controls are used in other studies performed by the group. Furthermore, looking at differences between our patient group and HC, the majority of the healthy controls in the current sample are young and well-functioning, with a lower BMI than the patients, possibly underestimating the impact of lifestyle factors on dysregulation of some inflammatory markers. The smoking status is not available for our healthy controls. However, comparing inflammatory markers within the patient population, only vWF was significantly affected with higher levels in smokers. Also, while the effect of smoking on inflammation is less documented, the effect of smoking on CVD risk is known. Therefore, since we had smoking status in our patients, we were able to adjust for smoking when evaluating CVD risk. Future studies should include control groups that are better matched with the patient group in regard to their metabolic status. Finally, in our study, only 14 patients were using lipid-lowering agents, thus our sample was statistically underpowered to address the issue if these agents can be used as an early intervention to reduce CVD risk.

## Conclusion

Patients with psychotic disorders have a markedly higher CVD risk compared to healthy controls as reflected by atherogenic lipid ratios, and the increased risk is associated with elevated hs-CRP and MPO reflecting subclinical inflammation and abnormal neutrophil activation in such patients. These findings support that low-grade inflammation may cause increased CVD risk in patients with psychotic disorders. These mechanisms should be further examined to determine the potential for treatment targets or development of novel risk evaluation strategies.

## Data Availability Statement

The datasets presented in this article are not readily available because sharing of data to external parties has not been approved by the ethics committee. Requests to access the datasets should be directed to e.j.reponen@medisin.uio.no.

## Ethics Statement

The studies involving human participants were reviewed and approved by Regional komite for medisinsk forskningsetikk, Øst-Norge (REK 1). The patients/participants provided their written informed consent to participate in this study.

## Author Contributions

All authors contributed to the article and approved the submitted version. Each author specifically made the following contributions to this manuscript: ER: Data collection, literature search, statistical analysis, and manuscript editing. ID: Data collection, literature search, statistical analysis, and manuscript editing. MT: Data collection, literature search, statistical analysis, and manuscript editing. RM: Data collection, statistical analysis, and manuscript editing. MA: Data collection, statistical analysis, and manuscript editing. TV: Data collection and manuscript editing. EH: Data collection and manuscript editing. OD: Data collection and manuscript editing. NS: Data collection and manuscript editing. SH: Data collection, literature search, and manuscript editing. AS: Literature search and manuscript editing. SMG: Literature search and manuscript editing. KW-R: Literature search and manuscript editing. SD: Data collection, literature search, and manuscript editing. IM: Data collection and manuscript editing. PA: Data collection, literature search, and manuscript editing. OA: Data collection, literature search, statistical analysis, and manuscript editing. TU: Data collection, literature search, statistical analysis, and manuscript editing.

## Funding

This work was supported by the Research Council of Norway (grant numbers 213837, 223273, 217776), the South-Eastern (grant numbers 2017-112, 2016-064) and Western (grant number 91141) Norway Regional Health Authorities, and the KG Jebsen Stiftelsen (grant number SKGJ-2011-36).

## Conflict of Interest

OA and RM have received speaker’s honorarium from Lundbeck. OA is consultant to HealthLytix.

The remaining authors declare that the research was conducted in the absence of any commercial or financial relationships that could be construed as a potential conflict of interest.
